# Comments on the Editor Re: Shi, Zumin, et al. “High Chili Intake and Cognitive Function among 4582 Adults: An Open Cohort Study over 15 Years.” *Nutrients* 2019, *11*(5), 1183

**DOI:** 10.3390/nu11122877

**Published:** 2019-11-26

**Authors:** Yun Wang, Dan Wu

**Affiliations:** 1 Department of Medicine, Washington University School of Medicine, St. Louis, MI 63110, USA; 2 Department of Clinical Research, London School of Hygiene & Tropical Medicine, London WC1E 7HT, UK

We read with great interest the article [1] in the June issue which warns of the health harms of chili intake from the 15-year study of 4582 Chinese adults. We felt keen to discuss the potential bias and limitation of this research, which would potentially donate some fact-bending findings.

First, a significant alarming issue is the reference to “chili peppers”. The authors defined them as “fresh and dried chili peppers, but not including sweet capsicum or black pepper”. However, the authors failed to notice the difference in dried and processed chilis versus the fresh chili. Fresh chili is more nutrient-rich due to bioactive ingredients, including capsaicin, vitamin C, and other nutrients such as vitamins A, K, and B6, and potassium [2]. Secondly, according to the authors, “dietary intake data” was collected by “conducting a 24 h dietary recall on each of 3 consecutive days”, and “Food and condiments in the home inventory, food purchased from markets or picked from gardens, and food waste were weighed and recorded by interviewers”. This is quite questionable based on the Chinese sharing dining culture. Separate dining is common in western culture, but in this study sample, a grouped dining style should be more common due to Chinese culture [3]. Therefore, it was less likely for the respondents to describe the exact quantity of the chili they were actually intaking from a sharing table. Thirdly, the cognitive screening that was included in this study was the composite scores of memories, counting back and subtraction score. It bears mentioning that the majority of the sample were poorly educated, therefore creating a challenge in their capacities to recall a 10-word list, or count backward from 20. The significant loss in the follow-up sample is also a drawback. The authors only included the participants who “completed at least one cognitive screen test” and very easily introduced the possibility of “reverse causality”, namely that the authors ruled out the people with mild-to-wild cognitive impairments who could not complete the cognitive tests due to the severity of cognitive impairments.

Additionally, there have been a lot of other confounding variables which would lead to the variation in cognitive decline, despite the adjustment in this research for intake of fat, smoking, alcohol drinking, income, urbanicity, education, physical activity, dietary patterns, BMI and hypertension. For example, the uptake of some medications (anticholinergic, psychoactive drugs, antidepressants and anticonvulsants [4]), some cardiovascular diseases (stroke [5]) and neurological diseases can contribute to cognitive decline but they are not considered in the study.

In summary, the self-reported nature of the data and the missing confounding variables can hardly make this analysis comprehensive and reliable to underline an association between chili consumption and cognitive decline.
